# E-Cigarette Use: Device Market, Study Design, and Emerging Evidence of Biological Consequences

**DOI:** 10.3390/ijms222212452

**Published:** 2021-11-18

**Authors:** Hunter T. Snoderly, Timothy R. Nurkiewicz, Elizabeth C. Bowdridge, Margaret F. Bennewitz

**Affiliations:** 1 Department of Chemical and Biomedical Engineering, Benjamin M. Statler College of Engineering and Mineral Resources, West Virginia University, Morgantown, WV 26506, USA; htsnoderly@mix.wvu.edu; 2 Center for Inhalation Toxicology, School of Medicine, West Virginia University, Morgantown, WV 26506, USA; tnurkiewicz@hsc.wvu.edu (T.R.N.); ebowdrid@hsc.wvu.edu (E.C.B.); 3 Department of Physiology and Pharmacology, School of Medicine, West Virginia University, Morgantown, WV 26506, USA

**Keywords:** e-cigarette, vaping, e-liquid, EVALI, ROS, epithelium, endothelium, platelets, macrophage, neutrophil

## Abstract

Electronic cigarettes are frequently viewed as a safer alternative to conventional cigarettes; however, evidence to support this perspective has not materialized. Indeed, the current literature reports that electronic cigarette use is associated with both acute lung injury and subclinical dysfunction to the lung and vasculature that may result in pathology following chronic use. E-cigarettes can alter vascular dynamics, polarize innate immune populations towards a proinflammatory state, compromise barrier function in the pulmonary endothelium and epithelium, and promote pre-oncogenic phenomena. This review will summarize the variety of e-cigarette products available to users, discuss current challenges in e-cigarette study design, outline the range of pathologies occurring in cases of e-cigarette associated acute lung injury, highlight disease supporting tissue- and cellular-level changes resulting from e-cigarette exposure, and briefly examine how these changes may promote tumorigenesis. Continued research of the mechanisms by which e-cigarettes induce pathology benefit users and clinicians by resulting in increased regulation of vaping devices, informing treatments for emerging diseases e-cigarettes produce, and increasing public awareness to reduce e-cigarette use and the onset of preventable disease.

## 1. Introduction

Electronic cigarettes (EC) are widely viewed by the public as a safer alternative to traditional cigarettes. While the existing body of literature has yet to conclusively establish the relative risk of ECs compared to conventional cigarettes, abundant evidence shows that EC use promotes concerning processes at the tissue, cellular, and biochemical scales. Some demonstrable EC aerosol (ECA)-induced changes include increased susceptibility to pulmonary infection, subclinical blood vessel changes similar to those observed in patients with vascular disease, and cellular dysregulation indicative of inflammation. Whether long-term EC use will promote pathology remains unclear. Additionally, the impact of any EC-specific pathology is likely compounded by the fact that nicotine itself is a cardiovascular toxicant, alters immune cell function, and is highly addictive. As such, the popularity of EC use is a substantial public health challenge. Therefore, it would be in the best interest of patients for clinicians and researchers to discuss and disseminate the risks of EC use to better enhance patient education. If chronic EC use promotes disease, targeted education may reduce its adoption and future impact. Characterizing the acute biological effects immediately following EC use will be beneficial in defining pathology associated with chronic EC use. Dialog between researchers and clinicians will be vital in uncovering and eventually treating these pathologies. This review discusses current techniques used and challenges faced by EC research, the potential for ECs to promote pulmonary and cardiovascular disease, ECA-induced changes to key cell populations, and how ECs may contribute to cancer.

## 2. EC Studies: Flavor and Device Market, Populations of Interest, Preclinical Study Design, and Current Challenges

There are a variety of factors impeding understanding of the pathologic effects produced by ECA exposure. Human observational studies are limited in their clinical utility by the vast commercial variety of available EC device designs and by difficulty in accounting for differences in use patterns between EC users. Furthermore, there is immense variability in commercially available aerosolized solutions (hereafter referred to as “e-liquids”), which are comprised of various compositions of propylene glycol (PG), vegetable glycerin (VG), and flavorants. In human studies, ECA inhalation patterns can vary substantially, as usage patterns are heterogenous [1]. EC users can instinctively modulate their behavior, including puff frequency, puff duration, and intervals between EC sessions to achieve their desired nicotine exposure levels. Smokers transitioning to ECs modulate their usage patterns to recapitulate the nicotine delivery profile of cigarettes; this differs from EC users with no prior history of smoking [2,3]. Therefore, properly controlling for the variation present across all facets of EC use is challenging in clinical studies. However, delineating the effects of this variability will be vitally important in unraveling the pathologic effects of EC use. Similar limitations exist in animal models of EC use including a lack of standard ECA exposure protocols, mode of delivery, difficulties in characterizing effective dose, whether clean air is cycled in between puffs, selecting an appropriate device model, and selecting appropriate e-liquid characteristics (i.e., PG/VG ratio, flavorants, and nicotine presence). These factors can dramatically influence the actual dose deposited in the lungs even when utilizing similar puff frequencies, making cross-study comparison much more challenging [4]. This review highlights the heterogeneity in ECA composition and generation mechanisms as well as key topics of interest. Current methods of exposure and their limitations in animal studies are also discussed.

### 2.1. Variability in Device Design

Substantial market diversity exists in EC device design. Fundamentally, these operate similarly. Every model includes certain key components including an e-liquid storage tank or pod, power source, heating element, and a means of producing aerosol at the e-liquid/heating element interface. Each of these components has the potential to modulate ECA generation in ways that may bear clinical relevance. For instance, a combination of battery longevity and e-liquid capacity may influence length and/or frequency of an EC user’s sessions. Similarly, the thermal and electrical properties of heating elements can vary substantially, resulting in a variety of operating temperatures. Higher temperatures increase the production of toxic carbonyl compounds including formaldehyde, acetaldehyde, and acrolein [5]. Moreover, a variety of species of toxic metallic nanoparticles are generated across different heating element brands and designs [6]. Due to their small size, metallic nanoparticles can easily penetrate through the airway tree, avoid mucociliary clearance, and deposit deep in the alveoli where they can cause inflammation in lung parenchyma and be systemically absorbed [7].

ECs can be subcategorized based on general design principles as follows: first generation ECs (or “cigalike” devices) mimic the appearance of cigarettes, are frequently disposable, store relatively little e-liquid, and are inefficient in their nicotine delivery [8,9]. Second generation devices are rechargeable, enable the user to replace or refill cartridges, and permit effective cumulative delivery of nicotine, similar to cigarettes [10,11]. Third generation devices offer the most flexibility, allowing a user to control wattage and temperature in addition to e-liquid composition; the tanks used in these devices are typically larger, refillable, and match the nicotine delivery profile provided by traditional cigarettes [11]. The array of options available in these devices allows users to adjust EC use to their preference and casts further complexity on studies seeking to characterize ECA exposure. For instance, two users operating the same device with the same e-liquid may be exposed to substantially different toxicant profiles depending on their temperature or power delivery settings. Generating ECA under both higher constant temperature and higher constant wattage conditions is known to increase the presence of free radicals in ECA [12]. Moreover, higher temperature or power settings, regardless of the mode of operation, decrease ECA particle size distribution [13].

The newest, most popular EC brands, such as Juul, blu, Vuse, and NJOY, do not clearly fit into the previously mentioned device generations, and could be considered to constitute the fourth generation ECs. These devices typically rely on replaceable prefilled pods/cartridges, although both blu and NJOY offer fully disposable models. The e-liquid within these pods/cartridges contains nicotine salts that are less irritating and deliver nicotine at much higher levels than previous EC generations [14] and even traditional cigarettes [15]. Though not endorsed by manufacturers, all of these pods/cartridges can also be refilled with other e-liquids for additional use [16]. However, to our knowledge, the prevalence of this practice amongst users has never been investigated, much less the effects this has on toxin release as a result of the increased taxation on the heating element after repeated refills. This behavior is analogous to the variability of coil replacement frequency in third generation devices, which also has toxicologic consequences; the longer a coil is in contact with e-liquid, the more heavy metals are released in ECA [17].

### 2.2. Variability in E-Liquid Formulation

In 2014, there were over 7500 unique e-liquid flavors listed for purchase online, with an average increase of 242 new flavors per month [18]. This diversity has reached even greater heights since then, with one survey based in the Netherlands reporting nearly 20,000 unique e-liquid formulations [19]. These products are highly unregulated until recently, and can vary widely in chemical composition and nicotine concentration [20], with flavor and nicotine preferences differing substantially amongst user populations. E-liquid classically consists of a base combination of PG and VG to generate an aerosol and carry added flavoring chemicals and nicotine [21]. Concern of toxicologic effects due to reactions between aldehyde-containing flavorings and PG or VG moieties are well documented, and result in the presence of formaldehyde, acrolein, and acetals in the inhaled ECA [22,23,24]. These have the capacity to cause inflammation, impair DNA repair [25], and produce cytotoxicity in airway cells [26]. Jensen et al. [27] showed that ECs utilizing high voltage settings in particular produced dramatically more formaldehyde hemiacetal, which are known as aldehyde-releasing agents that form during the decomposition of PG in the presence of VG. No formaldehyde hemiacetal was detected when operating ECs at lower voltage settings. Another study examining formaldehyde hemiacetal release claimed that these conditions only occurred in higher wattage conditions that resulted in coil overheating, which appeared to be ameliorated by increased e-liquid wicking material. EC users were able to detect and avoid these formaldehyde hemiacetal-producing conditions, as they resulted in foul tasting ECA [28]. Other studies have since established that free aldehydes are indeed produced during regular use conditions, although formaldehyde hemiacetal production increases dramatically with battery output [29,30]. In addition to PG/VG decomposition, flavorant degradation is a major contributor to aldehyde production [23,24]. Collectively, these results demonstrate the importance of accounting for device design, actual user practices during EC use, and e-liquid composition.

Flavoring chemicals alone can also contribute to cell death, impaired mitochondrial function, and inflammation [31,32,33]. The frequent lack of disclosure regarding specific flavoring ingredients by e-liquid manufacturers complicates investigation of the effects of such chemicals amongst users. Grana et al. [34] reported in their 2014 analysis of 59 ECA retail websites that tobacco and mint flavors were the most common varieties being offered, followed closely by fruit and candy flavors. Vanillin, maltol, benzaldehyde, ethyl acetate, cinnamaldehyde, citral, acetoin, diacetyl, and pentanedione are a small sampling of flavoring compounds known to be present in e-liquid. Though some of these compounds are generally recognized as being safe for consumption in food, mounting evidence suggests this is not the case in the context of EC use. While cytotoxicity measurements involving direct oral ingestion routes are not immediately translatable to the cytotoxicity of ECA, e-liquid ingredients such as ethyl maltol, cinnamaldehyde, and diacetyl are up to 100 times more concentrated in e-liquid compared to their use as food additives [26]. These extremely high concentrations prior to aerosolization, coupled with the delicate microenvironment of the lung, raise alarming implications for the toxicity of inhaled ECA. Compounding these concerns, hundreds of additional chemicals of potential toxicologic effect have also been detected [32,35,36,37].

Nicotine concentrations are as variable as concentrations of other e-liquid constituents. Although manufacturers typically report these metrics on-label (unlike flavorings), chromatographic analysis has revealed nicotine concentrations may vary up to 30% from reported concentrations [37,38,39]. Inaccurate nicotine reporting may cause users to modulate their EC use such that they are unknowingly exposed to higher overall quantities of ECA. For example, underreported nicotine concentration could cause users to unintentionally increase their total e-liquid consumption in the short term in order meet the demands of their nicotine dependency. Conversely, overreported nicotine concentrations may result in deepened overall dependency and more frequent EC use. Furthermore, the force and depth of inhalation by conventional cigarette smokers increases with nicotine dependency [40]. Though this phenomenon has yet to be investigated in EC users, it is conceivable that deeper inhalation would draw ECA and associated toxicants deeper into the lung.

Additionally, the efficiency of nicotine delivery appears to be modulated by choice of EC device style. For instance, one study showed that users of third generation devices achieved higher concentrations of nicotine plasma more rapidly than users of second generation devices, despite using e-liquid with lower nicotine content. Accordingly, users of third generation ECs consumed e-liquid more rapidly [11]. Nicotine salts used in fourth generation devices may also have altered pharmacodynamics and acute effects relative to e-liquids containing free-base nicotine (conventionally used in e-liquids). For instance, Shao and Friedman [41] found that nicotine salt-based e-liquid had a more acidic pH (protonated) relative to more basic conventional (unprotonated) e-liquid; they postulated that this reduces systemic bioavailability because protonated nicotine has poorer membrane permeability. Conversely, the authors suggested that protonated nicotine is more likely to result in acute lung damage, as this is the form that binds to nicotinic acetylcholine receptors (which activate inflammatory immune responses) lining the lung epithelium. These receptors are also expressed on macrophages; accordingly, alveolar macrophage production of inflammatory cytokines is enhanced in the presence of nicotine-containing ECA condensate compared to nicotine-free ECA condensate [42]. Thus, while nicotine’s toxicity is well established, understanding its behavioral and toxicologic effects as they relate specifically to EC use is critical in any broader discussion of the biological consequences of ECA inhalation.

Finally, EC users are known to create their own “at home” e-liquid blends. Though the frequency which users engage in this practice is unclear, this adds another layer of complexity to account for when studying EC-mediated effects. One small survey of users who engaged in this practice showed that approximately 25% of these individuals’ e-liquids contained a >20% difference between intended and actual nicotine content. Additionally, two chemicals of toxicologic concern, benzaldehyde and acetoin, were present in nearly half of all user-blended e-liquid samples studied [43].

### 2.3. Current Regulatory Proceedings

The United States Food and Drug Administration (FDA) has since taken steps to mitigate the startlingly diverse, youth-appealing, and underregulated EC and e-liquid market. In 2016, the FDA classified EC and e-liquid products as tobacco products and required that Premarket Tobacco Product Applications (PMTAs) be submitted for each e-liquid and EC component by late 2020. Thus far, the FDA has denied PMTAs affecting over 1 million flavored EC products. These applications failed to adequately demonstrate that they would ensure the protection of public health, specifically regarding the requirement that each product offer a greater benefit to cigarette smokers than risk posed to youth. A notable exception to this was the Vuse device with Vuse replacement tobacco flavor e-liquid filled pods. In conjunction with this, 10 other Vuse PMTAs for other flavored EC products were denied. Decisions are pending for EC products from other popular brands, most notably Juul and NJOY [44]. Increased regulatory scrutiny of ECs may result in a highly homogenous e-liquid market, which could provide a focused path for EC researchers as they seek to model actual use conditions. Additionally, the prevalence of “at home” e-liquid blending, which is already poorly studied, may increase amongst subsets of EC users should flavored e-liquids become widely unavailable. In short, questions surrounding the toxicologic effects and downstream pathologies resulting from specific EC styles and e-liquid flavorants will continue to be of interest as more longitudinal data are acquired to establish safety.

### 2.4. ECA Inhalation Study Design

In addition to the variety of devices and flavors available, researchers must choose the type of ECA exposure model, each having its own advantages and disadvantages, as summarized in Table 1. Models range from (1) human studies with EC users to (2) whole body rodent exposure systems with a nebulizer or EC device attached to (3) cell culture exposure with direct e-liquid contact or ECA delivery in an air–liquid interface configuration.

### 2.5. Human ECA Exposure Studies: Populations of Interest

While most challenging to execute and analyze, clinical studies of EC use in different populations will provide the greatest insight into the relevant pathological effects of ECA exposure on the pulmonary and cardiovascular systems. As ECs were initially designed as a harm reduction-based alternative to aid in enabling conventional cigarette users to quit smoking, there is a critical need to examine how effective ECs have been in their intended purpose as a smoking cessation tool. In fact, a systemic review of studies on smokers using ECs in conjunction with conventional cigarettes found that EC users had lower overall odds of quitting smoking than did non-users [45]. Accordingly, a more recent meta-analysis showed that EC use was only associated with quitting smoking when the device was used at least daily, and when provided as part of a smoking cessation clinical trial, rather than when used as a consumer product in observational studies [46]. In contrast, another recent study found that daily EC use was actually associated with poorer odds of quitting smoking in smokers seeking treatment [47]. Additionally, smokers using ECs who do successfully quit retain higher rates of nicotine dependency than those quitting with the assistance of drug-based therapies or non-EC nicotine replacement therapies [48]. Overall, it appears that the utility of ECs in smoking cessation is tenuous at best.

Despite the original intent behind their design, ECs have seen unprecedented adoption amongst youth, with a large subset of these individuals being non-smokers. The devices used by adolescent populations differ from adults, with youth less likely to use disposable devices mimicking cigarettes, instead favoring newer designs [49], which deliver nicotine more quickly and efficiently than previous device designs [11]. Despite this, several longitudinal studies and meta-analyses have shown that EC users are more likely to eventually start smoking cigarettes or increase their cigarette consumption [50,51,52]; however, these data must be examined critically. For instance, Levy et al. [53] found in their recent meta-analysis that overall cigarette use decreased substantially in youth around the time EC use became popular (which the authors define as 2014). This suggests that, while the risk of smoking initiation may exist in some users, ECs are more widely viewed among youth as an alternative to cigarettes. Regardless, the need to study pathologic effects of EC use in adolescent populations is clear.

Enhanced understanding of differences in usage patterns between new and more experienced EC users would facilitate better experimental design for studies distinguishing between acute and chronic exposure responses. The division between the two is already blurred; Shao et al. [54] describe a “chronic” exposure as 12 h exposures consisting of two episodes per hour, for 12 weeks. The authors classified the exposure paradigm as chronic because it recapitulated the nicotine pharmacokinetics seen in EC users. In contrast, Laube et al. [55] considered a “chronic” response to consist of one 20 min episode per day for three weeks, whereas the authors’ “acute” model consisted of one week of exposure. Though the definitions of “acute” and “chronic” are likely to be tissue and response specific, the utility of such terms is currently limited, and will remain so until individual pathologies are characterized over broader timespans.

### 2.6. Rodent ECA Exposure Studies

As an alternative to human studies, whole body exposure systems have been developed and are widely employed in the literature to facilitate the controlled exposure of rodents to ECA. However, this approach does have some limitations beyond what has been noted in Table 1. For instance, some ECA is deposited on the chamber and thus is lost. High fidelity measurements are required to fully define the dosing of animals undergoing exposure: aerosol concentration at the output, pulmonary deposition, and biomarkers indicative of exposure should all be defined. Despite this, most existing literature fails to examine metrics at each of these levels. Additionally, the presence of ECA condensate deposited on the animal itself may result in unintentional oral exposure due to grooming that occurs during and after exposures. Ocular deposition may represent another unintentional exposure pathway in whole body exposure systems. To our knowledge, no studies have examined the extent to which indirect oral or ocular exposure impacts effective ECA dose or whether either of these exposure pathways alters physiologic response to ECA in meaningful ways. However, some discussion of the possible consequences of oral or ocular exposure exists within the literature, although this is beyond the scope of this review [56,57]. Regardless, it is important to note that nosecone-based exposure systems intrinsically require other complicating factors to be introduced, as their use generally entails either anesthesia or physical restraint.

The lack of a standardized ECA exposure paradigm represents a significant hurdle to the translatability of such studies to clinical applicability. A common metric by which animal EC studies define their exposure is using “puffs”, which is typically based on the amount a user would inhale at one time; however, this practice is far from standardized, with puff duration and frequency varying substantially [55,58,59]. This contrasts strongly with research on conventional cigarettes, for which puff volume, duration, and frequency are internationally standardized [60]. The National Institute on Drug Abuse’s release of the Standard Reference E-Cigarette is an important step in addressing this deficiency [61,62]. However, standardized exposure methodologies modeling varying degrees of chronic and acute ECA exposure for both clinical and animal models are still critically needed.

## 3. Pulmonary Effects of EC Use

Numerous case studies and observational studies have produced results suggesting that e-liquid contributes to lung pathology in humans. This can occur as a direct response to specific toxicants such as diacetyl or Vitamin E acetate, adsorption of gaseous components of ECA, or deposition of solid particulate throughout the lung. ECA can trigger inflammation within the lung tissue and pulmonary vasculature, leading to immune cell recruitment, phenotypic alterations at the tissue and cellular scale, and further inflammatory response. In addition to initiating acute illness under certain circumstances, there is mounting evidence that chronic ECA exposure can also produce subclinical damage that may increase the risk of eventual adverse outcomes.

### 3.1. Acute Lung Injury

Perhaps the most well-known complication of EC use is E-cigarette, or vaping, associated lung injury (EVALI), which received widespread media coverage throughout 2019. To date, nearly 3000 EVALI cases have been reported in the United States [63]. Patients diagnosed with EVALI initially present with pneumonia-like symptoms; these symptoms progressively worsen and expand to include chest pain and hypoxia, sometimes culminating in death. Vitamin E acetate appears to be the most commonly causative agent for this acute illness. A recent study found that bronchoalveolar lavage (BAL) fluid in 94% of EVALI patients contained clinically relevant quantities of Vitamin E acetate [64]. Additionally, most of these patients had used e-liquids containing tetrahydrocannabinol (THC), the primary psychoactive component of marijuana; Vitamin E acetate happens to be commonly applied in THC containing products as a thickening agent [63,64].

Specific pathologies associated with EVALI have been documented in EC users, including exogenous lipoid pneumonia, which is characterized by the buildup of lipids in the lungs, and hypersensitivity pneumonitis, defined by inflammation and swelling of the lung tissue [65,66]. Lipoid pneumonia manifests as ground glass opacities in radiography as a hazy whiteness over areas of the lung on X-ray and computed tomography (CT) images and the presence of enlarged alveolar macrophages containing lipid in BAL fluid [66]. Some case reports have noted that lipid buildup is localized in the lung interstitium, or the space between the endothelium of the pulmonary capillaries and the alveolar epithelium; this phenomenon is accompanied by the infiltration of immune cell populations including neutrophils and macrophages in the interstitial space, where they provoke chronic inflammation [67,68]. Because symptoms may be mild or absent [69], the incidence of lipoid pneumonia amongst EC users is unknown. Whereas lipoid pneumonia is a macrophage-dominant pathology, hypersensitivity pneumonitis appears to be mediated by neutrophil recruitment, and is characterized by nodular, ground glass, or airspace opacities on X-ray/CT and the presence of pleural effusion, which is the buildup of fluid between the lungs and chest [65]. Elevated eosinophils may be present in BAL fluid, and predominance may occur with disease progression [70].

Bhat et al. [71] recently developed an animal model to mimic EVALI by exposing mice to inhaled aerosols generated from Vitamin E acetate. A substantial number of lipid-laden macrophages were observed in the BAL fluid and in lung histology sections from exposed mice using oil red O lipid staining. It is notable that, in this animal model, lipid-laden macrophages were also observed in mice exposed to ECA without Vitamin E acetate, albeit to a lesser extent than after exposure to ECA containing Vitamin E acetate. This suggests that, although the processes that lead to full-blown EVALI may be facilitated in the presence of Vitamin E acetate, normal EC use may activate these same pathways to a lesser degree over time. Should this prove to be the case, the implications are troubling for those engaging in long-term EC use, as these findings hint at the possibility of subclinical damage that may eventually lead to chronic disease.

Knowledge of specific causative agents of ECA-related pathologies is largely limited to observational case studies in EC users; few animal models exist that study specific acute etiologies seen in critically ill patients. Typically, organ-scale pathology is observed in hospitalized EC users via traditional clinical imaging methods, whereas animal studies typically define damage patterns through analysis of cytokine profiles, oxidative stress, or response of specific immune populations. Thus, there is a great need to conduct research that bridges the gap between the lab and the clinic to facilitate better understanding of the cellular and molecular basis for pathologies observed in patients.

### 3.2. Susceptibility to Infection and Chronic Damage Patterns

In addition to observations that EC use can produce primary disease, there is evidence that ECA inhalation may promote bacterial colonization of the lungs. For instance, Sussan et al. [58] demonstrated that mice exposed to nicotine-containing ECA possessed impaired bacterial clearance and exhibited increased weight loss. Additionally, lower survival upon infection with influenza was noted, indicative of dysfunction in multiple pathogen response mechanisms. Exposure was conducted over a period of two weeks, with each exposure occurring twice a day for 1.5 h. BAL fluid from ECA exposed mice infected with *Streptococcus pneumoniae* contained higher bacterial titers than BAL fluid from mice subjected to sham inhalation. The tendency for e-liquid to reduce macrophage phagocytosis was confirmed by the authors, suggesting that EC use may promote infection by impairing this response. Similarly, Miyashita et al. [72] found that mice exposed to ECA exhibited enhanced adhesion of *S. pneumoniae* in the airway, with further adhesion potentiated by nicotine. Notably, mice exposed to ECA expressed higher levels of platelet activating factor receptor (PAFR), a receptor utilized by *S. pneumoniae* when adhering to cells in the nasal epithelium. Furthermore, this effect was recapitulated in EC users, where PAFR expression was transiently increased immediately following use; however, this effect was not persistent, as baseline PAFR expression was similar between EC users and non-smokers. Regardless, this finding is concerning considering other phenotypic changes resulting from EC use that may promote infection. For instance, chronic ECA exposure in mice is associated with decreased mucociliary clearance [55], which is responsible for removing bacteria from the airways. Defective clearance is thus associated with greater susceptibility to infection and occurs in other disease states such as cystic fibrosis [73].

Though epidemiological studies examining whether EC users are at higher risk for respiratory infections are currently lacking, some preliminary studies suggest this may be the case. Sanou et al. [74] recently showed that incidence rates of respiratory infections are higher amongst U.S. Armed Services members who use EC compared to nonsmoking and smoking members. This effect appeared to be exacerbated in those who smoked and used ECs, as this group had the highest rates of respiratory infections. Another study showed that COVID-19 rates were positively and significantly correlated to the prevalence of EC use, but not conventional cigarette use, across U.S. state populations [75]. While this is not definitive evidence that EC use increases COVID-19 risk, this warrants additional study. In particular, EC use is thought to increase expression of the angiotensin-converting enzyme 2 receptor within the lung, which acts as the gateway for SARS-CoV-2 cellular infection [76].

Aside from reducing the capacity of the lungs to fight off pathogens, there is evidence that ECA also promotes an inflammatory state and altered morphology in the lung. Reinikovaite et al. [77] found that exposing rats to ECA resulted in the damage and subsequent enlargement of alveolar airspaces and capillary destruction, which is associated with emphysema. The extent of this damage was comparable to that caused by conventional cigarettes. The authors suggest that solid particles present in ECA may contribute to this pathology (rather than a specific component of ECA itself). Emphysema has also been observed in mice exposed to ECA, consistent with the presence of enlarged alveoli [78]. Additionally, aerosolized nicotine alone has enhanced inflammation, cell death, and pulmonary edema in rat lungs [79]. Cirillo et al. [80] showed that exposing rats to ECA over 28 days, even when operating ECs at modest power settings, promotes oxidative stress and lung inflammation in a manner consistent with chronic obstructive pulmonary disease (COPD). Specifically, features of this pathology included breakdown of alveoli and epithelial barrier function, evidence of apoptotic and necrotic cell presence, and dysregulated cytokine production similar to COPD such as upregulated IL-1β and IL-6. Another study found similar results in mice after four months of exposure [81]. Concerningly, a correlation between COPD and EC use has been noted across patient age groups even when accounting for other confounding variables such as conventional cigarette use, second-hand smoke exposure, use of other tobacco products, drug use, body mass index, physical activity, and demographic characteristics [82,83,84,85]. Collectively, this provides strong evidence that long-term EC use contributes to the development of chronic lung disease.

## 4. Cardiovascular Effects of EC Use

After ECA inhalation, toxic substances, their metabolites, and particulate matter promote cardiovascular dysfunction including increased arterial stiffness, angiogenesis, and alterations in blood flow and oxygenation. These hemodynamic changes increase the risk of cardiovascular disease in EC users. In an acute study by Shi et al. [86], increased collagen expression was noted in murine hearts after 14 days of ECA exposure containing nicotine. While this increase was not statistically significant, this raises the question of whether chronic EC use could produce cardiac fibrosis due to long-term exposure. Hearts of exposed mice were enriched in tissue markers of angiogenesis, which also occurred in kidney sections; this observation of enhanced angiogenesis is contrary to the decreased angiogenesis observed with cigarettes.

In addition to promoting angiogenesis, ECA inhalation has been linked to stiffer arteries, altered vascular reactivity and oxygenation, atherosclerotic plaques, and heart failure. By exposing mice to ECA over the course of eight months, Olfert et al. [78] directly showed that long-term EC use promotes altered vascular reactivity. Arterial stiffness was increased substantially, with no significant difference between arterial stiffness in EC-exposed and cigarette-exposed animals. Increased aortic stiffness ultimately results in increased blood pressure and reduced stroke volume, thereby increasing the risk of cardiovascular disease. Vascular reactivity to methacholine, a vasodilator, was also compromised in ECA and cigarette exposed animals compared to controls, indicating chronic ECA exposure promotes impaired endothelium-dependent dilation in arteries. In humans, Caporale et al. [87] also showed altered hemodynamics upon exposure to ECA. This study used magnetic resonance imaging to illustrate that even healthy non-smokers experienced impaired arterial dilation, decreased blood flow velocity and oxygenation in the superficial femoral artery and vein, respectively, after inhalation of nicotine-free ECA, illustrating that ECA can rapidly alter vessel hemodynamics. Notably, Carnevale et al. [88] showed that flow-mediated dilation, a marker of human endothelial function, for which lower values are associated with coronary artery disease, was reduced after EC use in both smokers and non-smokers similar to conventional cigarette smoking.

Utilizing a chronic model of exposure, another study used ECA exposed Apolipoprotein-E knockout mice (ApoE^−/−^; a murine atherosclerosis model) to examine ECA-induced cardiovascular pathology after three months of ECA with and without nicotine [89]. Interestingly, early development of systolic heart failure occurred only in the presence of nicotine, with increased atherosclerotic plaque buildup and altered signaling evident in pathways involving inflammation, circadian rhythm, and leukocyte extravasation. However, this study also found significant differences in gene expression between mice exposed to aerosolized saline and non-nicotine ECA; thus, non-cardiac effects of nicotine-free EC use cannot be ruled out. Although it remains to be seen whether EC use mediates atherosclerotic plaque development in humans, Boas et al. [90] previously demonstrated that EC use modulated the splenocardiac axis, in which the spleen releases proinflammatory monocytes that travel to and destabilize arterial plaques, ultimately resulting in ischemia. Thus, even if EC use does not directly result in increased plaque formation, it may pose a risk to users who have these conditions due to other factors. In addition, daily EC use is independently associated with myocardial infarction risk, albeit not to the extent of cigarette smoking [91]. Ongoing research on the effect of ECs on cardiovascular health must dissect the link between the outcomes seen in acute versus chronic studies, as the mechanisms by which responses to acute exposure generate long-term pathology remain unclear but seem unlikely to be identical.

## 5. Cellular Effects of EC Use

To facilitate further understanding of lung and cardiovascular pathologies associated with EC use, there is a critical need to systematically characterize the resulting damage on the cellular level. Many questions remain as to whether the mechanisms behind the observed effects of EC use are similar to those involved in cigarette smoking. While increased oxidative burden appears to be a common feature of both tobacco smoke and ECA inhalation [92,93,94], unique pathologies caused by e-liquid specific components, such as lipoid pneumonia [66], complicate direct comparison. Understanding the effects of e-liquid exposure on specific cell populations (Table 2) will be critical to unraveling the mechanisms which drive these pathologies.

### 5.1. Oxidative Stress

Markers of increased oxidative stress have been noted in subjects exposed to either ECA or conventional cigarettes alone; however, this has not been consistent across e-liquid compositions or exposure durations. Oxidative stress causes damage to the cell membrane and cellular components including proteins, lipids, and DNA through the generation of free radicals and results in a constitutively activated inflammatory state. These reactive oxygen or nitrogen species (ROS or RNS) include superoxide (O_2_^−^), hydrogen peroxide (H_2_O_2_), hydroxyl radicals (OH), and nitric oxide (NO). Glynos et al. [103] found that after acute (defined as occurring over three days) exposure to ECA containing both nicotine and flavoring, mouse lung homogenates and BAL fluid displayed increased levels of oxidative stress and pro-inflammatory interleukins IL-6 and IL-1β. Interestingly, however, this was not observed in mice receiving ECA containing nicotine only without flavoring; these mice instead exhibited similar cytokine profiles and lower oxidative stress burden relative to mice exposed to conventional cigarette smoke. Other studies have confirmed that acute exposure to ECA generates increased oxidative stress in mice [58,92]. Similar trends have been observed in human EC users. Reidel et al.’s [104] recent study that collected sputum from both chronic smokers and EC users found the expression of oxidative stress response proteins to be similarly elevated in both populations relative to non-smokers. Thus, it is not surprising that one particular EC type was found to contain 7 × 10^11^ free radicals per each 2 s puff, as opposed to 10^14^ radicals per puff in a standard research cigarette [58,105]; free radicals are well known to cause oxidative stress when they outnumber antioxidants and can explain some of the damage caused by ECA exposure. Similarly, Lerner et al. [31] showed differences in ROS intrinsically present in ECA from varying e-liquids via a cell free ROS assay. ROS presence varied with flavor and nicotine content, with menthol flavoring producing more ROS than tobacco flavoring. Oxidative stress was reduced in devices with lower nicotine content. ROS present in ECA invoke inflammation, ultimately resulting in increased oxidative stress [94,104,106].

### 5.2. Epithelial Cells

During EC use, epithelial cells lining the airways are the first cell type to encounter the chemicals and free radicals contained in ECA. When exposed to e-liquid, epithelial cells have been shown to upregulate expression of pro-inflammatory cytokines such as IL-6, IL-8, IL-15, IL-17, IL-1β, and IFNγ; this response seems to occur across a variety of e-liquid compositions [31,32,97]. Increased presence of chemotactic factors, such as platelet derived growth factor, granulocyte colony stimulating factor, and granulocyte-macrophage colony stimulating factor, was also observed; these mediate epithelial damage by excessively recruiting pro-inflammatory white blood cells. Furthermore, e-liquid exposure has been noted to impair epithelial cell function, including decreases in ciliary motility, mitochondrial respiration, ion transport, and membrane resistance [95,96,97]. Accordingly, ECA exposure enhances necrosis and apoptosis in airway epithelial cells [107]. Compromise of epithelial cells in the airway increases susceptibility to infectious disease and paves the way for further inflammation to occur as epithelial cells release neutrophil and macrophage recruiting factors. The duration of these effects, whether they occur chronically or transiently, and whether they eventually result in persistent epithelial dysfunction and tissue damage warrants further study.

### 5.3. Endothelial Cells

After encountering the lung epithelium, substances in ECA may cross into the capillaries surrounding the alveoli to result in abnormal endothelial cell function and generation of ROS in the lung vasculature. Nicotine is known to have a dose dependent effect on endothelial permeability; however, e-liquid mediated endothelial barrier dysfunction does not appear to be solely nicotine-dependent [93]. Compromised barrier function is conducive to the extravasation of inflammatory immune cells seen in EC-associated lung pathologies. Relative to cigarette smoke, ECA appears to have less of a cytotoxic effect on endothelial cells, though it is still associated with increased cell death when compared to sham exposure. Anderson et al. [108] found that human umbilical vein endothelial cells exposed to high doses of ECA extracts experienced both apoptotic and necrotic cell death; this effect is ROS-dependent, but was not fully eliminated by antioxidant treatment. The oxidative stress generated by e-liquid in the absence of nicotine is thought to be mediated by the presence of acrolein, which can also form in unflavored e-liquid. Acrolein activates nicotinamide adenine dinucleotide phosphate (NADPH) oxidase in blood vessels, thus resulting in nicotine-independent ROS production [109]. This effect has also been confirmed in patients, with notable upregulation of NADPH oxidase in smokers using EC, albeit to a lesser extent than that caused by cigarettes [88]. Ikonomidis et al. [110] showed that oxidative stress could ultimately be reduced in smokers partially switching to ECs over the course of one month, with higher oxidative stress positively correlating with the total number of cigarettes smoked during the transition.

### 5.4. Platelets

While in the bloodstream, toxic chemicals and ROS generated from ECA can interact with circulating blood cells including platelets which are responsible for coagulation. In fact, thrombocytopenia is a known complication of EVALI, in which a low platelet count is observed in the blood. One reason why platelet count could drop with EVALI is due to increased activation and sequestration of platelets in the blood vessels due to EC use. Although nicotine alone inhibits platelet function, ECA has been observed to be a potent platelet activator. A recent study [111] collected platelets from 50 non-smoker volunteers and exposed them to ECA extract and cigarette smoke extract with or without nicotine, finding that aggregation was increased for all groups. Increased activation was also noted regardless of nicotine concentration, suggesting that ECA itself was the causative agent of platelet hyperactivity.

Nocella et al. [112] conducted a study on platelet adhesion molecules in EC users including both smokers and non-smokers. Interestingly, the authors found that non-smokers experienced a significant increase in soluble P-selectin and the pro-inflammatory CD40 ligand (both adhesive ligands enabling platelet/leukocyte interaction and platelet activation) in as little as 5 min after EC use. While this effect was not seen in smokers who had a higher baseline expression of P-selectin and CD40 ligand, enhanced platelet activation after EC use was noted for all groups. There is additional evidence that platelets can release small particles called extracellular vesicles expressing P-selectin or CD40 into the blood plasma after EC use, and these vesicles are increased in non-smokers exposed to nicotine containing ECA relative to those exposed to nicotine-free ECA [99].

Platelet activation following ECA exposure has been corroborated in animal models. Following an acute whole-body ECA exposure protocol in mice, platelet aggregation and granule secretion were increased with an upregulation in integrin expression upon isolation; tail bleed clotting time was reduced when compared to mice exposed to sham inhalation protocol [59]. Similarly, a study exposing mice to ECA from a Juul device using menthol flavored e-liquid over two weeks reduced tail bleed time [100]. Upon exposure to adenosine diphosphate or thrombin, platelets harvested from ECA exposed mice underwent aggregation at higher rates than did healthy platelets. It is notable that this effect occurred despite no significant net increase in platelet counts, suggesting that ECA potentiates functional changes in platelets. In line with this finding, the authors of this study observed that ECA exposed platelets generated more P-selectin. Whether the apparently enhanced thrombotic state observed after EC use increases the risk of acute pathologic consequences remains unclear, but this merits further study.

### 5.5. Macrophages

Two myeloid-lineage populations can be affected by ECA: resident macrophages in the alveoli and recruited monocytes from the blood that cross the blood–air barrier and differentiate into macrophages in the air spaces in response to the inflammatory lung environment produced by EC use. Incubation of macrophages with flavored and unflavored e-liquid in vitro results in increased IL-6, IL-8, MMP-9 and TNFα expression, DNA damage, oxidative stress, apoptosis, and necrosis compared to controls. This suggests that EC use provokes a strong inflammatory response [42,97]. The presence of lipids within alveolar macrophages from patients with ECA-induced lipoid pneumonia further supports that e-liquid exposure directly alters macrophage phenotype [66]. ECA with or without nicotine appears to impair macrophage phagocytosis [42,95], which as mentioned previously can negatively impact bacterial clearance and encourage pulmonary infection. Furthermore, macrophage clearance of apoptotic/necrotic epithelial cell remains is impaired upon exposure to ECA [107]. Taken together, macrophage-mediated inflammation and matrix remodeling promote further epithelial dysfunction, resulting in a positive feedback loop in which inflamed and damaged tissues release chemotactic signals that further recruit monocytes from the blood to the area of insult. Macrophages and monocytes also mediate dysfunction in the interior of blood vessels by promoting the development of ECA-induced atherosclerotic plaques via toll-like receptor 9 activation; blockade of this receptor ameliorated the production of inflammatory cytokines in the plasma [113].

### 5.6. Neutrophils

Given the inflammatory milieu of cytokines and neutrophil-recruiting factors elicited from other cells in and adjacent to the circulation, it would seem intuitive that ECA enhances neutrophil activation in a similar manner to macrophages. When activated, neutrophils are recruited from the bloodstream to extravasate into the alveolar spaces in response to EC exposure. However, there is seemingly conflicting evidence as to whether e-liquid promotes or inhibits neutrophil activation and subsequent inflammatory response. In fact, the data suggest that this may depend largely on e-liquid constituency. For example, Corriden et al. [114] found that neutrophil chemotaxis, neutrophil extracellular trap formation (NETosis, or release of fibrous DNA), phagocytosis and ROS production were all impaired in neutrophils exposed ex vivo to unflavored ECA with nicotine compared to unstimulated neutrophils; these processes were also inhibited in neutrophils exposed to both ECA and phorbol myristate acetate (PMA), a potent inducer of classical NETosis and neutrophil activation, versus PMA alone. In vivo, lower neutrophil response was observed in EC-exposed mice challenged with *S. pneumoniae*, as a result of impaired chemotaxis. However, these suppressive effects were not observed when exposing neutrophils to nigericin, which activates a noncanonical, alternative NETosis pathway; thus, neutrophils may contribute to ECA-induced inflammation in the absence of bacterial infection if ECA activates NETs via a noncanonical signaling pathway. Markers of NETosis, including MMP-9 and NE, have been detected at higher concentrations in the lungs of EC users [94,102,104], supporting that neutrophils can also be activated by EC use. Clapp et al. [95,101] further clarified that the observed effect of ECA on neutrophil phenotype may be dependent on flavoring compounds, as they observed that cinnamaldehyde increased NETosis both on its own and in the presence of PMA relative to other flavors. Although cinnamaldehyde decreased neutrophil phagocytosis, other flavors, such as cola, did not alter phagocytosis significantly. Thus, it seems likely that the reduced efficacy of neutrophil pathogen response, including phagocytosis, contributes to the increased susceptibility to respiratory bacterial colonization. In contrast, ECA may also enhance neutrophil inflammatory response, which could result in lung dysfunction by enhancing NETosis, promoting endothelial damage, and provoking the formation of occlusive and pro-inflammatory neutrophil/platelet aggregates. Further work will be needed to clarify the extent to which ECA induces a shift in neutrophil polarity from antimicrobial to pro-inflammatory.

**Figure 1:**
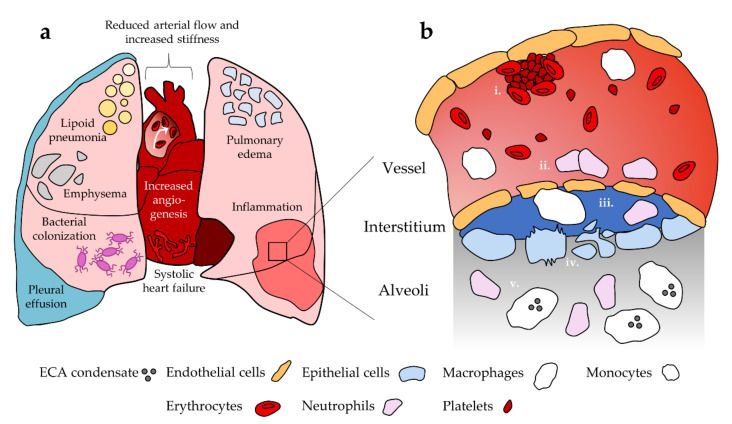
Summary of pulmonary and cardiovascular dysfunction and their cellular basis. (**a**) EC use has been linked to disease and dysfunction in the heart and lungs. (**b**) i. Platelets are activated towards a pro-thrombotic phenotype in the vessel; ii. ECA exposure promotes endothelial cell death and compromised barrier function, which facilitates immune cell extravasation into the surrounding tissue. Endothelial cells release inflammatory cytokines that enhance neutrophil recruitment; iii. Monocytes differentiate into macrophages upon extravasation. Neutrophils and macrophages remodel extracellular matrix in the interstitium as an inflammatory response, promoting compromise of the epithelium and endothelium; iv. Epithelial permeability is compromised and ECA exposure is associated with epithelial cell apoptosis and necrosis; v. ECA exposed macrophages and neutrophils enter the lung tissue and promote inflammation. Macrophages take up EC-associated lipids. Not to scale.

Recent evidence also suggests that neutrophil response to ECA in mice is sex dependent. To assess sex differences, Wang et al. [115] exposed male and female mice to aerosolized PG with and without nicotine. BAL fluid from female mice exposed to PG with nicotine contained significantly elevated neutrophil count relative to groups exposed to room air control and PG alone. No significant increase was observed in male mice for any group. Furthermore, levels of myeloperoxidase activity in BAL fluid, indicative of neutrophil oxidative stress and NETosis, were comparable between males and females; while myeloperoxidase activity was significantly enhanced with PG alone, the addition of nicotine had a suppressive effect. Thus, it is possible that exposure to PG/VG aerosols without nicotine may also lead to neutrophil-mediated lung damage. This observed sex difference introduces another level of complexity in studying neutrophil response to ECA, and no studies have examined whether this occurs in human users. Furthermore, it is unknown whether healthy neutrophils behave differently versus neutrophils in EC users or animal models that have been repeatedly exposed to ECA. Neutrophils maturing in the presence of microenvironmental changes induced by chronic ECA exposure may respond differently to ECA inhalation relative to neutrophils that are not habituated to these conditions. Figure 1 summarizes the role of neutrophils and other cellular populations of interest after ECA exposure as well as pulmonary and cardiovascular disease and dysfunction.

## 6. Cancer and EC Use

EC use promotes a variety of processes relating to the hallmarks of cancer and across the stages of disease progression [116]. While initial research has detected a decreased presence of carcinogen metabolites in the urine of EC users versus smokers [117], ECA still contains carcinogens and may be capable of supporting tumorigenesis [118]. However, it is currently unclear whether ECA predominantly promotes tumorigenesis directly, enhances primary tumor growth and survival, supports metastasis, or acts at all stages of cancer. While several case studies have reported oral cancer development in patients with a history of EC use [119,120], long-term epidemiological studies are critically needed to establish whether EC users experience higher rates of cancer.

There is no evidence to support the use of ECs as a smoking cessation tool in patients diagnosed with cancer [121]. As ECs provide no benefit for enabling smoking cessation amongst this population and promote effects that may contribute to tumor progression, it would seem prudent that clinicians educate patients on these risks and discourage EC use. Further study of whether long-term EC use initiates or enhances carcinogenic processes is of critical need to prevent an epidemic of ECA-induced malignancies.

Canistro et al. [122] showed in rats that ECA exposure induces pro-inflammatory, carcinogenic pathways such as those modulated by the cytochrome P450 superfamily. Cytochrome P450 enzymes can promote toxic effects through drug metabolism, activation of pre-mutagens and pre-carcinogens, and production of ROS. Thus, it is not surprising that ECA exposure resulted in a significant increase of cytochrome P450 enzymes and free radicals in rat lungs together with a reduction in antioxidant enzymes. Systemically, ECA exposure led to genotoxic effects in rat blood and urine; single and double stranded DNA breaks were observed in peripheral blood leukocytes while base pair substitutions and frame shift mutations were detected in *S. typhimurium* bacteria incubated with rat urine from exposed animals using the Ames test. Interestingly, the Ames test utilizing ECA condensate in vitro has been shown to be mutagenic [123,124], suggesting that ECA may cause DNA damage and mutagenesis both directly and indirectly as the result of increased oxidative stress and by reducing DNA repair activity in pulmonary and cardiovascular tissues [25].

ECA contains carcinogenic compounds that enhance ROS formation, promote inflammation, and contribute to aberrant expression of oncogenic growth factors. Given that the lag time for malignancy development following the initiation of chronic use is expected to be around 20 years for cigarette smokers, we may have yet to see the true long-term effects of ECs [125]. As such, no studies have been conducted which examine rates of cancer diagnosis amongst chronic EC users. However, there are many potential pathways by which ECs contribute to phenotypic changes known to be pro-oncogenic in nature. EC use results in the production of ROS [93] that can contribute to DNA damage, and has been linked to double stranded DNA breaks and repair inhibition [25].

**Figure 2:**
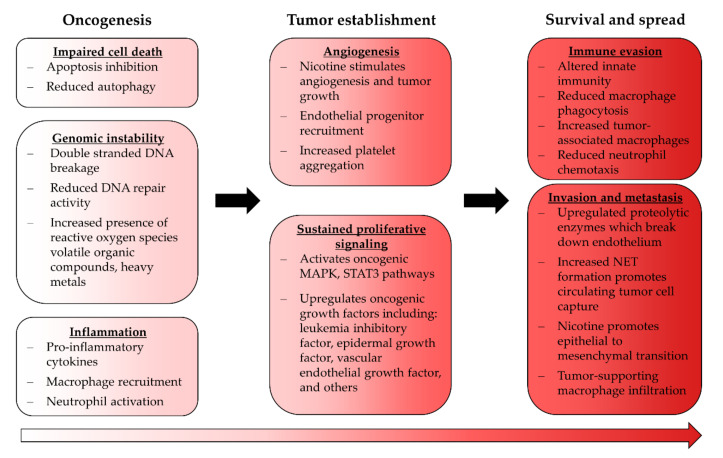
Effects of ECA exposure relating to cancer. Some ECA-induced phenotypical changes are consistent with tumor-supporting processes and may indicate that EC use carries a risk of carcinogenesis; however, the severity of this risk has yet to be established.

EC use is known to promote several pro-oncogenic phenomena that may support continuing tumor development after initiation (Figure 2). Furthermore, EC use upregulates leukemia inhibitory factor which activates the MAPK and STAT3 pathways, both of which are well established oncogenic signaling pathways; whether ECA modulates these in the context of cancer has not been investigated [126]. Nicotine itself promotes these pathways in endothelial cells, which can reduce apoptosis and impair autophagy, both of which are critical steps in tumor establishment [127,128,129]. ECA also appears to be proinflammatory, which fosters oncogenic transformation when sustained [103,104].

There is more evidence that EC use supports the growth and immune evasion of existing malignancies. For instance, ECA supports angiogenesis, which if occurring in a tumor, would facilitate the ongoing growth and survival of already established malignancies [101,130]. Recently, Pham et al. [131] produced conclusive evidence that EC use may aid in sustaining malignancy. Immunocompetent mice undergoing orthotopic breast tumor cell injection exposed to nicotine-containing ECA successfully developed tumors in all cases, whereas only one third of the control group did. Furthermore, the size doubling time of these tumors was increased two-fold relative to unexposed mice. Mice exposed to ECA also exhibited more tumor-associated macrophage infiltrate compared to the control group, which suggests that ECA exposure polarizes macrophages towards a tumor-supporting phenotype. Finally, ECA exposed mice experienced significantly higher metastatic burden in the lungs. While this study is limited by a relatively small sample size and its failure to distinguish the contributing role of nicotine in these outcomes, it clearly illustrates that EC use is of particular concern in users susceptible to or diagnosed with cancer.

The tumor-supporting potential of ECA is further supported by Huynh et al.’s study [132] showing that ECA exposed NOD-SCID-Gamma mice receiving tail vein injections of breast cancer cells experienced greater lung colonization and lower tumor cell apoptosis than did mice not exposed to ECA. It has been demonstrated that ECA caused epithelial to mesenchymal transition in one lung cancer cell line; this effect is essential for tumor cells to invade the circulation and begin to establish metastases [133]. If this phenomenon also occurs in other cancers, this may explain the apparent pro-metastatic effects of ECA exposure. Additionally, it is well established that platelet aggregation is enhanced by ECA exposure [100,111,112]; platelets also tend to “cloak” circulating tumor cells to protect them from immune detection and aid in circulating tumor cell adhesion to the endothelium. The latter effect may be responsible for the enhanced tumor cell colonization in ECA exposed mice in this study.

## 7. Conclusions

ECs represent an emerging health concern and their long-term impacts have yet to be fully elucidated. Based on the current published literature, evidence substantiating the notion that ECs are safer than conventional cigarettes does not exist. This appears to be due to the presence of toxicants in e-liquid composition, their adverse effects in animal models, association with acute lung injury and cardiovascular disease, and ability to modulate different cell populations in the lung and blood towards pro-inflammatory phenotypes. Continuing to research the mechanisms by which ECA induces cellular and organ level damage will highlight compelling evidence that can benefit both users and clinicians. Uncovering definitive pathways of EC-mediated injury could lead to increased regulation of the EC market, inform the development of novel treatments for emerging diseases that ECs may produce, and increase public awareness to reduce EC use and the onset of preventable disease. Large scale clinical research focusing on EC users and specific at-risk populations, as well as basic research unraveling the effects of ECA on a cellular and molecular scale, will be vitally important to these efforts.

## Figures and Tables

**Table 1:** Summary of general models to study ECA exposure and their advantages and disadvantages.

Type	Description	Advantages	Disadvantages
Human studies	Studies that examine EC use amongst never-smoker EC users, former-smoker EC users, non-users, or other groups of interest.	Detects evidence of acute and subclinical pathology	Difficult to control for device, e-liquid characteristics, and frequency of use
		Best physiologic and clinical relevance	Heavily dependent on subject compliance and accurate self-reporting
		Can observe EC use in populations of interest	Difficult to observe long-term outcomes due to relatively new EC popularity
Whole body or nosecone rodent exposure via nebulizer	Aerosol is generated by nebulizing e-liquid rather than via EC device.	Tight controlled overall ECA delivery	Lack of heating element reduces clinical relevance
		Highly homogenous individual ECA “puffs”	Homogenous ECA “puffs” do not correspond to actual use
		Enables the addition of labels to track cellular uptake and lung deposition	No standardized ECA exposure paradigm
Whole body rodent or nosecone exposure via EC	ECA is generated by either a whole EC device or through coil heating (similar to EC) and delivered to the animal in a manner comparable to actual use.	Closely mimics actual ECA delivery	Measuring variations in individual ECA “puffs” requires specialized equipment
		Realistically heterogenous individual ECA “puffs”	No standardized ECA exposure paradigm
		ECA delivery, device, and e-liquid selection can be tailored to study design	
Cell culture exposure via direct stimulation	E-liquid is added to cell culture media directly.	Precise control of dosage	Difficult to determine physiologically relevant dosages in vitro
		Rapid analysis of multiple e-liquid formulations on cells of interest	Lack of heating element reduces clinical relevance
		Does not require specialized equipment	Direct cell exposure to e-liquid does not model actual ECA exposure
Cell culture exposure via ECA	Cells are exposed to ECA generated by EC device.	Recapitulates actual ECA exposure in vitro	Air–liquid interface must be considered to accurately model ECA delivery
		Direct observation of ECA exposure on cells of interest	Specialized equipment required to expose multiple cultures in parallel
		ECA delivery, device, and e-liquid selection can be tailored to study design	

**Table 2:** Effects of varying device and e-liquid formulations on phenotype for selected cell populations. * indicates nicotine salt e-liquid. ↑ indicates increase; ↓ indicates decrease; --- indicates no change. Abbreviations listed in footer ^1^.

Cell Population Impacted	E-Liquid Components	Nicotine Level	Flavoring	E-Liquid Brand	Effects	In Vivo/In Vitro
Epithelial	PG and VG	16 mg/mL	Tobacco, commercial	Blu	↑ IL-6, ↑ IL-8 [31]	In vitro
	N/A	N/A	Acetoin, pentanedione, maltol, OR o-vanillin	N/A	↑ IL-8 [32]	In vitro
	55% PG, 45% VG	N/A	Cinnamon, commercial	Local	↓ Ion transport [95]	In vitro
	100% VG	1.10%	Tobacco, commercial	Johnson Creek	↓ Ciliary motility, ↓ Mitochondrial respiration [96]	In vitro
	50% PG, 50% VG	60.9 mg/mL *	Cucumber, commercial	Juul	↑ IL-8, ↑ IL-15, ↑ IFNγ, ↑ IL-17, ↑ PDGF, ↑ MCP-1, ↓ Membrane resistance [97]	In vitro
	50% PG, 50% VG	60.9 mg/mL *	Menthol, commercial	Juul	↑ IL-8, ↑ IL-15, ↑ IL-17, ↑ IL-1β, ↑ IFNγ, ↑ PDGF, ↑ MCP-1, ↑ G-CSF [97]	In vitro
	50% PG, 50% VG	60.9 mg/mL *	Mango, commercial	Juul	↑ IL-8, ↑ IL-15, ↑ IL-1β, ↑ IFNγ, ↑ PDGF, ↑ G-CSF, ↑ GM-CSF, ↑ Prostaglandin E2α [97]	In vitro
	50% PG, 50% VG	60.9 mg/mL *	Coffee, commercial	Juul	↑ IL-8, ↑ IL-15, ↑ IFNγ, ↑ PDGF, ↑ GM-CSF, ↑ Prostaglandin E2α [97]	In vitro
Endothelial	50% PG, 50% VG	24 mg/mL	N/A	N/A	↑ Angiogenesis, ↑ CD31, ↑ CD34, ↑ Capillary density [86]	In vivo
	PG and VG	24 mg/mL	Unspecified	Blu	↑ ROS, ↓ Membrane resistance, ↑ CD31, ↑ CD54, ↑ CD106 [98]	In vitro, in vivo
	50% PG, 45% VG, 5% ethanol	19 mg/mL	N/A	Valeo Laboratories	↑ P-selectin, ↑ Extracellular vesicle secretion [99]	In vivo
Platelets	30% PG, 70% VG	18 mg/mL	Menthol, commercial	Absolute Zero	↑ Granule secretion, ↑ Thrombogenesis, ↓ Occlusion time [59]	In vivo
	50% PG, 50% VG	60.9 mg/mL *	Menthol, commercial	Juul	↑ CD40, ↑ P-selectin, ↑ Granule secretion, ↑ Thrombogenesis, ↓ Occlusion time [100]	In vivo
	50% PG, 45% VG, 5% ethanol	19 mg/mL	N/A	Valeo Laboratories	↑ CD40, ↑ P-selectin, ↑ Extracellular vesicle secretion [99]	In vivo
Macrophages	50% PG, 50% VG	60.9 mg/mL *	Cucumber, commercial	Juul	↑ DNA damage [97]	In vitro
	50% PG, 50% VG	60.9 mg/mL *	Menthol, commercial	Juul	↑ Prostaglandin E2α, ↑ DNA damage [97]	In vitro
	50% PG, 50% VG	60.9 mg/mL *	Coffee, commercial	Juul	↑ IL-8, ↑ DNA damage [42]	In vitro
	50% PG, 50% VG	36 mg/mL	N/A	American E-liquids Store	↑ IL-6, ↑ IL-8, ↑ TNFα, ↑ MCP-1, ↑ MMP-9, ↑ ROS, ↑ Necrosis, ↑ Apoptosis [95]	In vitro
	55% PG, 45% VG	N/A	Cinnamon, commercial	Local	↓ Phagocytosis, ↓ IL-6, ↓ IL-8 [101]	In vitro
	55% PG, 45% VG	N/A	Cola, commercial	Local	--- Phagocytosis, ↑ IL-6 [101]	In vitro
Neutrophils	30% PG, 70% VG	18 mg/mL	Menthol, commercial	Absolute Zero	--- Activation [101]	In vivo
	55% PG, 45% VG	N/A	Cinnamon, commercial	Local	↓ Phagocytosis, --- IL-8, ↑ NETosis, ↑ NETosis w/ NET stimuli [101]	In vitro
	55% PG, 45% VG	N/A	Cola, commercial	Local	--- Phagocytosis, ↑ IL-8, --- NETosis, ↑ NETosis w/ NET stimuli [101]	In vitro
	PG and VG	24 mg/mL	Unspecified	VIP	↑ MMP9, ↑ IL-8, ↑ NE [102]	In vitro

^1^ PG—propylene glycol; VG—vegetable glycerin; IL—interleukin; IFNγ—interferon gamma; PDGF—platelet derived growth factor; MCP-1—monocyte chemoattractant protein-1; G-CSF—granulocyte-colony stimulating factor; GM-CSF—granulocyte macrophage colony-stimulating factor; CD—cluster of differentiation; ROS—reactive oxygen species; TNFα—tumor necrosis factor alpha; MMP-9—matrix metalloproteinase-9; NET—neutrophil extracellular trap; NE—neutrophil elastase.
